# On detour index of cycloparaphenylene and polyphenylene molecular structures

**DOI:** 10.1038/s41598-021-94765-6

**Published:** 2021-07-27

**Authors:** S. Prabhu, Y. Sherlin Nisha, M. Arulperumjothi, D. Sagaya Rani Jeba, V. Manimozhi

**Affiliations:** 1 Department of Mathematics, Sri Venkateswara College of Engineering, Sriperumbudur, 602117 India; 2 Department of Mathematics, Sri Sairam Institute of Technology, Chennai, 600044 India; 3 Department of Mathematics, Loyola College, University of Madras, Chennai, 600034 India; 4 Department of Mathematics, Panimalar Engineering College, Chennai, 600123 India

**Keywords:** Chemistry, Mathematics and computing, Nanoscience and technology

## Abstract

Cycloparaphenylene is a particle that comprises a few benzene rings associated with covalent bonds in the para positions to frame a ring-like structure. Similarly, poly (para-phenylenes) are macromolecules that include benzenoid compounds straightforwardly joined to each other by C–C bonds. Because of their remarkable architectural highlights, these structures have fascinated attention from numerous vantage focuses. Descriptors are among the most fundamental segments of prescient quantitative structure-activity and property relationship (QSAR/QSPR) demonstrating examination. They encode chemical data of particles as quantitative numbers, which are utilized to create a mathematical correlation. The nature of a predictive model relies upon great demonstrating insights, yet additionally on the extraction of compound highlights. To a great extent, Molecular topology has exhibited its adequacy in portraying sub-atomic structures and anticipating their properties. It follows a two-dimensional methodology, just thinking about the interior plan, including molecules. Explicit subsets speak the design of every atom of topological descriptors. When all around picked, these descriptors give a unique method of describing an atomic system that can represent the most significant highlights of the molecular structure. Detour index is one such topological descriptor with much application in chemistry, especially in QSAR/QSPR studies. This article presents an exact analytical expression for the detour index of cycloparaphenylene and poly (para-phenylene).

## Introduction

Nanomaterials, materials along highlights or sizes going from $10^{-9}$ m to $10^{-7}$ m in at least one measurements^1^ are the centre of a developing scientific insurrection. The primary favourable circumstances of these materials are organic, electronic, and mechanical properties not established in traditional materials^2,3^. Joining the particular interesting properties along their notable acknowledgment capacities^4^ has brought about systems with fundamentally improved execution^5^ and major applications across chemistry^6^, physics^7^, biology^8^, medicine^5,9^, and food technology^10^. Aside from huge mechanical quality and least weight, a large portion of nanomaterials’ remarkable attributes is connected to their surface properties^9^, which empower improved associations with numerous biological entities. Such communications depend on the size, manufacture system, and explicit calculation of the nanoparticles. As anticipated, these qualities joined along with the capacity to shape hydrogen bonds, scattering powers, stacking, dative bonds, and hydrophobic associations can influence the strength and selectivity of nanomaterials^11^. Subsequently, nanomaterials particular properties have started attention in analytical chemistry and must be utilized to create contemporary utilization in sample sensing, separation, and provision.

Carbon nanotubes (CNTs) promise to reform a few material science fields and are proposed to open the route into nanotechnology^12,13^. These circular rod-shaped carbon nanostructures have novel attributes that lead them to be conceivably valuable in numerous applications in nanoscience and nanotechnology. CNTs have pulled in noteworthy consideration due to their wonderful mechanical^14^, and electronic^15^. Structural consistency of the CNT is fundamentally significant as the sidewall structures (armchair and zigzag) decide huge numbers of the compelling properties of CNTs^13,16^. In a perfect world, scientists would incorporate CNTs with a characterized target sidewall structure and diameter. However, the current engineered techniques, for example, curve release and substance fume testimony, give CNTs a combination of different forms. Hence, the particular and unsurprising union of basically systematic CNTs would speak to a fundamental development in nanocarbon science, and chemistry^17^.

**Figure 1:**
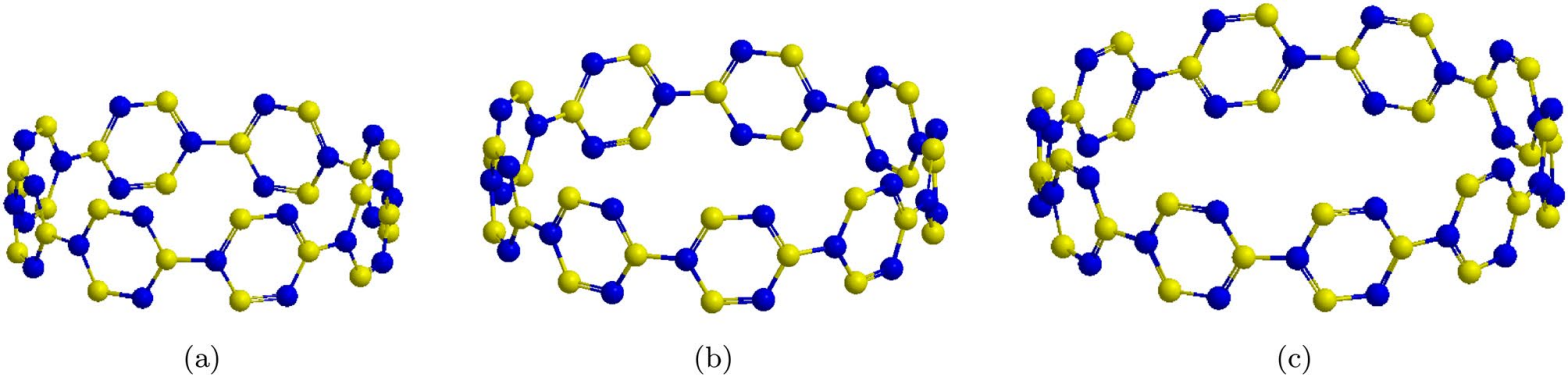
**(a)** $[8]-$CPP; **(b)** $[9]-$CPP; **(c)** $[10]-$CPP.

There are different kinds of carbon-based nanostructures such as carbon nanorings^18,19^, nanosprings^20^, and nanocones^21^, etc. Carbon nanorings have been seen in single-walled carbon nanotubes (SWCNTs) developed by laser vaporization^22^. The diameters of these round structures range amid 300 and 500 nm and their widths somewhere in the range of 5 and 15 nm. There are no topological pentagon-heptagon deserts in these structures, as kinks that could be made by such imperfections are not watched. In this manner, they can be imaged as the bowing of a straight SWCNT into a ring by associating its two closures to shape the carbon nanorings. These nanoring structures might be ideal nanodevices because of their interesting mechanical^13^, and physical properties^19^. Among the most limited formed piece of armchair carbon nanotubes (CNTs), cycloparaphenylenes (CPPs) have as of late pulled in expanding consideration from scientists. CPPs have straightforward hoop-shaped structures comprising aromatic rings with para-linkage, which were guessed 50 years ago, yet blended distinctly in the last decade^23^. Their stressed and contorted aromatic frameworks and radially arranged *p* orbitals have fascinated manufactured physicists, theoreticians, supramolecular scientific experts, and materials researchers the same. In spite of this boundless importance, the CPPs remain an difficult synthetic challenge. It is trying to make brilliant, stable CPPs with a little HOMO-LUMO gap because of restricting strain based reactivity and symmetry-based fluorescence extinguishing for little CPPs^24^. A few exploration bunches have created combinations of [*n*] CPPs of distinctive ring sizes (here *n* speaks to the quantity of benzene) as depicted in Fig.  1. Different techniques have incredibly researched the impact of [*n*]CPPs on the microelectronic stuff^25^. The sum of characteristic polynomial of [*n*] CPPs were reported in^26^.

**Figure 2:**
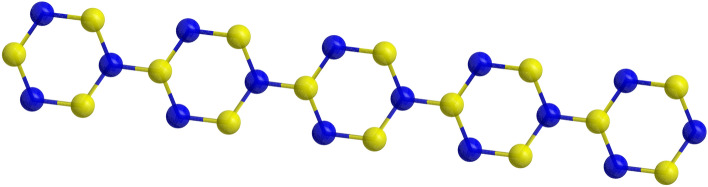
Polyphenylene.

Linear poly(para-phenylene) (PPP) are polymeric compounds with hexagonal rings as reproducing units as shown in Fig. 2 and are significant polycyclic aromatic compounds that are discovered to be the fundamental units of numerous novel materials like graphene or related compounds. Because of these, linear PPPs have been the focal point of fascination for both experimentalists and chemists^27–29^ since the most recent couple of many years. Linear PPPs and their derivatives have broadly been utilized in optoelectronic applications. Although there are a variety of linear polyaromatic polymers^30^, linear polyphenylenes are extremely insoluble unless they have solubilizing functional groups that can form hydrogen bonds with water. For instance, functional groups such as OH, NH$_2$, and COOH.

Topological indices (TIs) of huge chemical structures, for example, metal natural systems can be amazingly valuable in both portrayal of structures and processing their physicochemical properties that are generally difficult to calculate for such enormous organizations of significance in reticular chemistry. It is a mathematical quantity which bonds molecular topology to molecular properties^31^. Such entities are invariants of graph and are utilized as descriptors for QSAR/QSPR examinations^32,33^, proven to be an vigorous zone at the frontline of research. These descriptors are exceptionally valuable for looking through database of molecule, predicting molecular properties^34^, screening of drug^5^, designing of drug^9^, complex networks^35,36^ and many other procedures. The idea of this molecular descriptor came from Wiener’s effort in 1947^37^. He detected that there is a high degree of correlation amid the melting point with the Wiener index^38–40^.

## Background

Graph theory is a field of mathematics with potential application in engineering and science^41^. The theory provides a solid foundation for investigating topological requirements of many systems. Kaveh and Koohestani^42,43^ have effectively applied graph theory to the optimal analysis of FEMs in the framework of the force method in structural mechanics. Graph theory’s practical and beneficial applications include visualisation of sparse matrices, nodal ordering, envelope reduction, graph partitioning, and configuration processing. The reader who is interested can search up where the majority of these applications have been recorded in^44,45^. We use the theory to generate reducible representations of symmetry groups, taking into account the unique specifications of graphs.

Let |*V*(*G*)| and |*E*(*G*)| be the number of vertices and edges of a chemical graph *G* respectively. For any two vertices *x* and *y* are adjacent if there is an edge between them. Distance between two vertices $x, y \in V(G)$ is the number of edges in the shortest path connecting them in a connected graph *G* and is denoted by $d_G(x,y)$^46,47^. Similarly, the detour distance^48,49^ among two vertices $x, y \in V(G)$ is the number of edges in the longest path connecting them in a connected graph *G* and is denoted by $l_G(x, y)$. Also with the note $d_G(u,u)=0$ and $l_G(u,u)=0$, the *transmission* (farness or vertex Wiener value) of a vertex $u\in V(G)$ defined by *W*(*u*), as the sum of the lengths of all shortest paths between *u* to all other vertices in *G*^50–53^. Following this, we define the *detour transmission* (vertex detour value) of a vertex $u\in V(G)$ is denoted by $\omega (u)$ and explained as the sum of the detour lengths of all longest paths between *u* to all other vertices in *G*. Mathematically,

1 $$\begin{aligned} W(u)=\sum _{v\in V(G)}d_G(u,v), \end{aligned}$$

and

2 $$\begin{aligned} \omega (u)=\sum _{v\in V(G)}l_G(u,v). \end{aligned}$$

The Wiener index *W*(*G*) is the sum of shortest distance between every pair of vertices, where as the detour index $\omega (G)$ is the sum of longest distance between every pair of vertices^48,54,55^. Mathematically,

3 $$\begin{aligned} W(G)=\sum _{\{u,v\} \subseteq V(G)}d_G(u,v)=\frac{1}{2}\sum _{u\in V(G)}W(u), \end{aligned}$$

and

4 $$\begin{aligned} \omega (G)=\sum _{\{u,v\}\subseteq V(G)}l_G(u,v)=\frac{1}{2}\sum _{u\in V(G)}\omega (u). \end{aligned}$$

The application of detour index in QSAR considers is clarified by Lukovits in^56^. Rücker^57^ additionally researched this idea as a invariant for melting points of alkanes of cyclic and acyclic nature. It is noticed that Wiener index and detour index are equivalent if and only if *G* is acyclic and there are a few research papers on Wiener index of trees with a given condition and those result hold for detour index. It merits researching the detour index of cyclic graphs. For additional details on this investigation refer^58–61^.

In^60^ the authors derived an algorithm for recognizing the longest path among any pair of vertices of a graph and it was utilized to calculate an exact analytical formula for the detour index of fused bicyclic networks. Computer strategies for computing the detour distances and subsequently for calculating the detour index was derived in^54,57^. It has been demonstrated in^62^, and the detour matrix is a NP-complete problem. A strategy for building the detour matrix^63,64^ for graphs of modest sizes were introduced in^65^. Inter correlation amid hyper-detour index and other TIs such as Wiener, Harary, hyper-Wiener, hyper-Harary, and detour index were evaluated in^60^ on three pairs of branched and unbranched. Cycloalkanes and alkanes and with up to eight carbon particles and the hyper-detour index have been examined in structure-property studies^33^. Ongoing applications of the hyper-detour index discovered in^66^.

The detour index has also had great success combined with the Wiener index in structure boiling point modelling of cyclic and acyclic hydrocarbons. In^54^ the authors analyzed the importance of the detour index and correlated its application with the Wiener index. Also, they established that the detour index combined with the Wiener index is very adequate in the structure-boiling point modelling of acyclic and cyclic saturated hydrocarbons. This achievement has prompted the advancement of related indices such as the hyper-detour index^67^ and the Cluj-detour index^59^. Qi and Zhou^67^ presented the hyper-detour index of unicyclic graphs and decided the graphs with the smallest and biggest detour indices respectively in the class of *n*-vertex bicyclic graphs with precisely two cycles for $n\ge 5$. In^68^, Du decided the graphs with the second and the third smallest and biggest detour indices in the class of *n*-vertex bicyclic graphs with precisely two cycles for $n \ge 6$. Very recently Prabhu et al. have found the detour index for join of graph^69^. This paper presents an expression for the detour index of cycloparaphenylene and poly (*p*-phenylene) using detour transmission of a vertex.

## Results

In Ref.^61^, the experimental and calculated boiling points (°C) of 76 alkanes and cycloalkanes, as well as their Wiener and Detour indices, are reported. For acyclic structures, the Wiener index *W* and the detour index *ω* are, of course, identical. *W* and *ω* are not very intercorrelated indices for polycyclic structures. The linear correlation between *W* and *ω* (*ω* = *aW* + *b*) for a set of 37 diverse polycyclic graphs was presented with a modest correlation coefficient (*r* = 0.79) in Ref.^55^, while the exponential relationship between *W* and *ω* (*ω* = *aW*^b^) produced only a slightly better correlation between them (*r* = 0.86). With this motivation, we proceed to find the detour index of the CPP and PPP. In this section, we first explain the vertex set and edge set of cycloparaphenylene and poly (*p*-phenylene) before proceeding to our main results.

**Figure 3:**
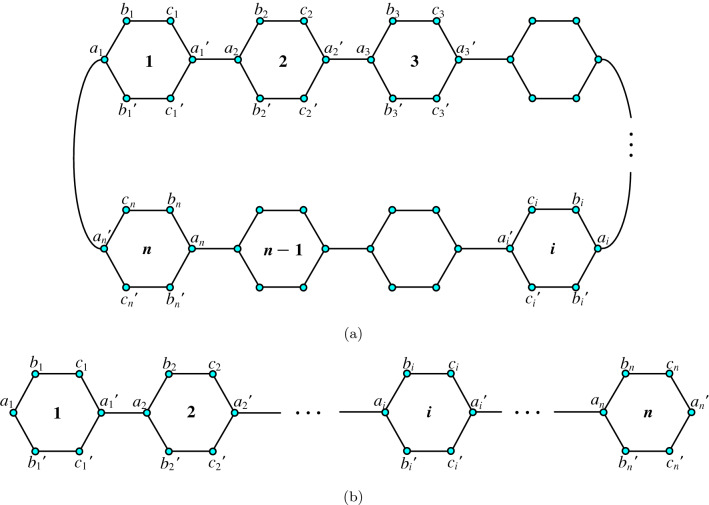
**(a)** Cycloparaphenylene $[n]-$CPP; **(b)** polyphenylene PPP(*n*).

It is observed from the molecular structure of cycloparaphenylene $[n]-$CPP and polyphenylene PPP(*n*) the vertex set of these two molecular graphs remains same and is given by $\{a_i,a_i',b_i,b_i',c_i,c_i': 1 \le i \le n\}$ with cardinality as 6*n*. The edge set for $[n]-$CPP is given by $\{a_ib_i, a_ib_i', b_ic_i, b_i'c_i', c_ia_i', c_i'a_i', : 1\le i \le n\} \cup \{a_i'a_j: 1\le i<j\le n\}$ with $|j-i|= 1$ or $n-1$ and for PPP(*n*), it is $\{a_ib_i, a_ib_i', b_ic_i, b_i'c_i', c_ia_i', c_i'a_i', : 1\le i \le n\} \cup \{a_i'a_j: 1\le i<j\le n\}$ with $|j-i|= 1$. And their cardinalities are respectively given by 7*n* and $7n-1$. The molecular graph of cycloparaphenylene and polyphenylene were depicted in Fig. 3a,b.

### **Lemma 1**

*Let* *G* *be a molecular graph of a cycloparaphenylene and* $\{a_i,a_i',b_i,b_i',c_i,c_i': 1 \le i \le n\}$ *be the vertex set of* *G*. *Then for any vertex* $a_i \in V(G)$,

$$\begin{aligned} l_G(a_{1},a_{i})=\left\{ \begin{array}{ll} 4(i-1) &{} \hbox {if }i >\lceil \frac{n}{2} \rceil \\ 4(n-i+1) &{} \hbox {if }i \le \lceil \frac{n}{2} \rceil \end{array} \right. \end{aligned}$$

### **Proof**

**Figure 4:**
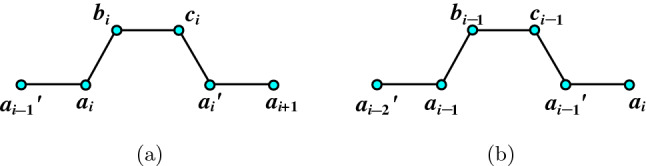
**(a)** Hamilton-path string of length $4(i-1)$; **(b)** Hamilton-path string of length $4(n-i+1)$.

For $i>\lceil \frac{n}{2} \rceil$, the set $\{a_{k}, b_{k}, c_{k}, a'_{k} : 1\le k \le i-1 \}$ induces a path of length $4(i-1)$. See Fig. 4a.

Clearly Fig. 4b depicts the path of length $4(n-i+1)$ for $i\le \lceil \frac{n}{2} \rceil$. $\square$

### **Lemma 2**

*Let* *G* *be a molecular graph of a cycloparaphenylene and* $\{a_i,a_i',b_i,b_i',c_i,c_i': 1 \le i \le n\}$ *be the vertex set of* *G*. *Then*,

(i) $$l_G(a_1,b_{i})= l_G(a_1,b'_{i})= \left\{ \begin{array}{ll} 4(n-i)+5 &{} \hbox {if }i \le \lceil \frac{n}{2} \rceil \\ 4i+1 &{} \hbox {if }i > \lceil \frac{n}{2} \rceil \end{array} \right. $$
(ii) $$l_G(a_1,c_{i})= l_G(a_1,c'_{i})= \left\{ \begin{array}{ll} 4(n-i)+6 &{} \hbox {if } i \le \lceil \frac{n}{2} \rceil \\ 4i &{} \hbox {if } i > \lceil \frac{n}{2} \rceil \end{array} \right. $$
(iii) $$l_G(a_1,a'_{i})=\left\{ \begin{array}{llll} 4(n-i)+1 &{} \hbox {if } i \le \lfloor \frac{n}{2} \rfloor \\ 4i-1 &{} \hbox {if } i > \lfloor \frac{n}{2} \rfloor \\ \end{array} \right. $$
(iv) $$l_G(b_1,a'_{i})=\left\{ \begin{array}{ll} 4(n-i)+6 &{} \hbox {if } i \le \lceil \frac{n}{2} \rceil \\ 4i &{} \hbox {if } i > \lceil \frac{n}{2} \rceil \end{array} \right. $$
(v) $$l_G(b_1,b_{i})= l_G(b_1,b'_{i})= \left\{ \begin{array}{ll} 4(n-i)+10 &{} \hbox {if }i \le \lceil \frac{n}{2} \rceil \\ 4i+2 &{} \hbox {if } i > \lceil \frac{n}{2} \rceil \end{array} \right. $$
(vi) $$l_G(b_1,a_{i})=\left\{ \begin{array}{ll} 4(n-i)+9 &{} \hbox {if } i \le \lceil \frac{n+1}{2} \rceil \\ 4i-3 &{} \hbox {if }i > \lceil \frac{n+1}{2} \rceil \end{array} \right. $$
(vii) $$l_G(b_1,c_{i})= l_G(b_1,c'_{i})= \left\{ \begin{array}{ll} 4(n-i)+11 &{} \hbox {if }i \le \lceil \frac{n+1}{2} \rceil \\ 4i+1 &{} \hbox {if }i > \lceil \frac{n+1}{2} \rceil \end{array} \right. $$

### **Proof**

**Figure 5:**
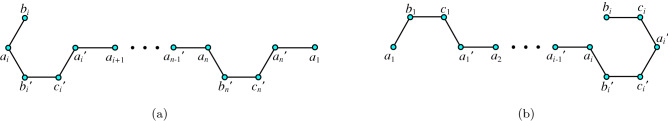
**(a)** Hamilton-path string of length $4(n-i)+5$; **(b)** Hamilton-path string of length $4i+1$.

**Figure 6:**
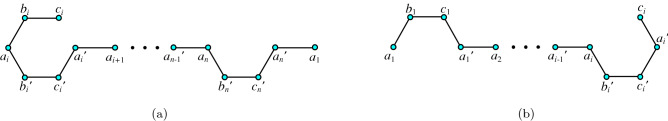
**(a)** Hamilton-path string of length $4(n-i)+6$; **(b)** Hamilton-path string of length 4*i*.

**Figure 7:**
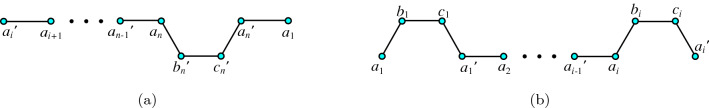
**(a)** Hamilton-path string of length $4(n-i)+1$; **(b)** Hamilton-path string of length $4i-1$.

**Figure 8:**
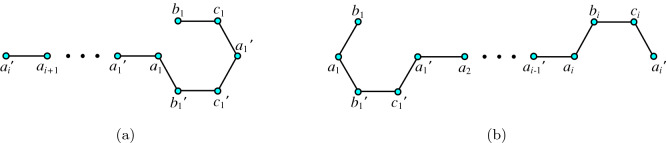
**(a)** Hamilton-path string of length $4(n-i)+6$; **(b)** Hamilton-path string of length 4*i*.

**Figure 9:**
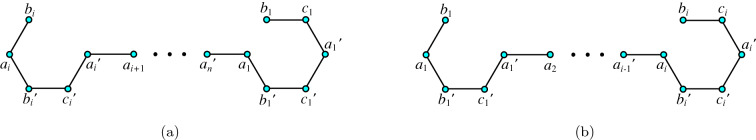
**(a)** Hamilton-path string of length $4(n-i)+10$; **(b)** Hamiltom-path string of length $4i+2$.

**Figure 10:**
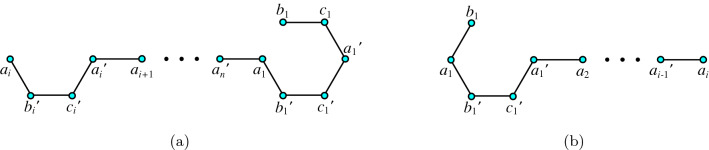
**(a)** Hamilton-path string of length $4(n-i)+9$; **(b)** Hamilton-path string of length $4i-3$.

**Figure 11:**
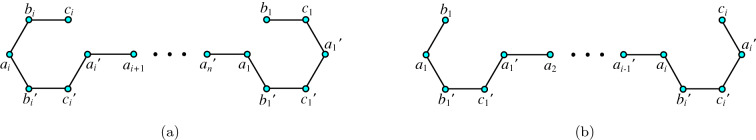
**(a)** Hamilton-path string of length $4(n-i)+11$; **(b)** Hamilton-path string of length $4i+1$.

(i) For $$i\le \lceil \frac{n}{2} \rceil $$, $$ l_G(a_1,b_{i})= l_G(a_1,a_{i})+l_G(a_i,b_i) =4(n-i)+5$$, and for $$ i>\lceil \frac{n}{2} \rceil $$, $$ l_G(a_1,b_{i})= l_G(a_1,a_{i})+l_G(a_i,b_i) =4i+1 $$, see Fig. 5.
(ii) For $i\le \lceil \frac{n}{2} \rceil$, $l_G(a_1,c_{i})= l_G(a_1,a_{i})+l_G(a_i,c_i) =4(n-i)+6$, and for $i> \lceil \frac{n}{2} \rceil$, $l_G(a_1,c_{i})= l_G(a_1,a_{i})+l_G(a_i,c_i) =4i$ as shown in Fig. 6.
(iii) For $i\le \lfloor \frac{n}{2} \rfloor$, $l_G(a_1,a'_{i})= l_G(a_1,a_{i+1})+l_G(a_{i+1},a'_i) =4(n-i)+1$. For $i > \lfloor \frac{n}{2} \rfloor$, $l_G(a_1,a'_{i})= l_G(a_1,a_{i})+l_G(a_{i},a'_i) =4i-1$. See Fig. 7.
(iv) For $i\le \lceil \frac{n}{2} \rceil$, $l_G(b_1,a'_{i})= l_G(b_1,a_{1})+l_G(a_{1},a_{i+1})+l_G(a_{i+1},a'_i) =4(n-i)+6$. For $i > \lceil \frac{n}{2} \rceil$, $l_G(b_1,a'_{i})= l_G(b_1,a_{1})+l_G(a_{1},a_{i})+l_G(a_{i},a'_i) =4i$. See Fig. 8.
(v) For $i\le \lceil \frac{n}{2} \rceil$, $l_G(b_1,b_{i})= l_G(b_1,a_{1})+l_G(a_{1},a_{i})+l_G(a_{i},b_i) =4(n-i)+10$. For $i > \lceil \frac{n}{2} \rceil$, $l_G(b_1,b_{i})= l_G(b_1,a_{1})+l_G(a_{1},a_{i})+l_G(a_{i},b_i) =4i+2$. See Fig. 9.
(vi) For $i\le \lceil \frac{n+1}{2} \rceil$, $l_G(b_1,a_{i})= l_G(b_1,a_{1})+l_G(a_{1},a_{i}) =4(n-i)+9$, and for $i > \lceil \frac{n+1}{2} \rceil$, $l_G(b_1,a_{i})= l_G(b_1,a_{1})+l_G(a_{1},a_{i}) =4i-3$. For details refer Fig. 10.
(vii) For $$i\le \lceil \frac{n+1}{2} \rceil $$, $$ l_G(b_1,c_{i})= l_G(b_1,a_{1})+l_G(a_{1},a_{i})+l_G(a_i,c_i) =4(n-i)+11 $$. For $$ i > \lceil \frac{n+1}{2} \rceil $$, $$ l_G(b_1,c_{i})= l_G(b_1,a_{1})+l_G(a_{1},a_{i})+l_G(a_i,c_i) =4i+1$$. The Hamilton-path construction is depicted in Fig. 11.

$$\square $$

The following lemma is straight forward from the structural property of [*n*]-CPP and the addressing scheme proposed in the begening of this section.

### **Lemma 3**

*Let* *G* *be a molecular graph of a cycloparaphenylene and* $\{a_i,a_i',b_i,b_i',c_i,c_i': 1 \le i \le n\}$ *be the vertex set of* *G*. *Then*,

(i) $$l_G(a_i,a'_{i})=4n-3.$$
(ii) $$l_G(a_i,b_{i})=l_G(a_i,b'_{i})= l_G(b_i,a_{i})= l_G(b_i,c_{i}) =l_G(b_i,c'_{i})= 4n-1.$$
(iii) $$l_G(a_i,c_{i})=l_G(a_i,c'_{i})= l_G(b_i,a'_{i}) =4n-2.$$
(iv) $$l_G(b_i,b'_{i})= 4n.$$

### **Lemma 4**

*Let* *G* *be a molecular graph of a cycloparaphenylene and* $\{a_i,a_i',b_i,b_i',c_i,c_i': 1 \le i \le n\}$ *be the vertex set of* *G*. *Then*,

(i) $$ \omega (a_1)=\left\{ \begin{array}{ll} 18n^2+8n-12 &{} \hbox {if }n\hbox { is even} \\ 18n^2+8n-13 &{} \hbox {if }n\hbox { is odd} \end{array} \right. $$
(ii) $$\omega (b_1)=\omega (c_1) =\left\{ \begin{array}{ll} 18n^2+26n-30 &{} \hbox {if }n\hbox { is even} \\ 18n^2+26n-29 &{} \hbox {if }n\hbox { is odd} \end{array} \right. $$

### **Proof**

For *n* even, the detour transmission of $a_1 \in V(G)$ is given by,

$$\begin{aligned} \omega (a_{1})&= \sum \limits _{x \in V(G)} l_G(a_{1},x)\\&= \sum \limits _{i=2}^{n} l_G(a_{1},a_{i}) +\sum \limits _{i=1}^{n} l_G(a_{1},b_{i}) + \sum \limits _{i=1}^{n} l_G(a_{1},c_{i}) + \sum \limits _{i=1}^{n} l_G(a_{1},a'_{i}) +\sum \limits _{i=1}^{n} l_G(a_{1},b'_{i}) + \sum \limits _{i=1}^{n} l_G(a_{1},c'_{i})\\&= \sum \limits _{i=2}^{n} l_G(a_{1},a_{i})+2 \sum \limits _{i=2}^{n} l_G(a_{1},b_{i})+ 2\sum \limits _{i=2}^{n} l_G(a_{1},c_{i})+ \sum \limits _{i=2}^{n} l_G(a_{1},a'_{i})\\&\quad +l_G(a_{1},a'_{1})+2l_G(a_{1},b_{1}) +2l_G(a_{1},c_{1})\\&= \sum \limits _{i=2}^{\frac{n}{2}} l_G(a_{1},a_{i})+\sum \limits _{i=\frac{n}{2}+1}^{n} l_G(a_{1},a_{i})+2\Bigg [\sum \limits _{i=2}^{\frac{n}{2}} l_G(a_{1},b_{i})+\sum \limits _{i=\frac{n}{2}+1}^{n} l_G(a_{1},b_{i})\Bigg ] + 2\Bigg [ \sum \limits _{i=2}^{\frac{n}{2}} l_G(a_{1},c_{i}) \\&\quad +\sum \limits _{i=\frac{n}{2}+1}^{n} l_G(a_{1},c_{i})\Bigg ] +\sum \limits _{i=2}^{\frac{n}{2}} l_G(a_{1},a'_{i}) + \sum \limits _{i=\frac{n}{2}+1}^{n} l_G(a_{1},a'_{i})+l_G(a_{1},a'_{1})+ 2l_G(a_{1},b_{1}) +2l_G(a_{1},c_{1})\\&= \sum \limits _{i=2}^{\frac{n}{2}} 4(n-i+1) +\sum \limits _{i=\frac{n}{2}+1}^{n} 4(i-1)+2\Bigg [\sum \limits _{i=2}^{\frac{n}{2}} [4(n-i)+5]+ \sum \limits _{i=\frac{n}{2}+1}^{n} (4i+1)\Bigg ] \\&\quad +2\Bigg [ \sum \limits _{i=2}^{\frac{n}{2}} [4(n-i)+6] +\sum \limits _{i=\frac{n}{2}+1}^{n} 4i\Bigg ]\\&\quad +\sum \limits _{i=2}^{\frac{n}{2}} [4(n-i)+1] + \sum \limits _{i=\frac{n}{2}+1}^{n} (4i-1) +(4n-3)+2(4n-1)+2(4n-2) \\&= 18n^2+8n-12. \end{aligned}$$

For *n* odd,

$$\begin{aligned} \omega (a_{1})&= \sum \limits _{x \in V(G)} l_G(a_{1},x)\\&= \sum \limits _{i=2}^{n} l_G(a_{1},a_{i}) +\sum \limits _{i=1}^{n} l_G(a_{1},b_{i}) + \sum \limits _{i=1}^{n} l_G(a_{1},c_{i}) +\sum \limits _{i=1}^{n} l_G(a_{1},a'_{i}) +\sum \limits _{i=1}^{n} l_G(a_{1},b'_{i}) + \sum \limits _{i=1}^{n} l_G(a_{1},c'_{i})\\&= \sum \limits _{i=2}^{n} l_G(a_{1},a_{i})+2 \sum \limits _{i=2}^{n} l_G(a_{1},b_{i}) + 2\sum \limits _{i=2}^{n} l_G(a_{1},c_{i}) +\sum \limits _{i=2}^{n} l_G(a_{1},a'_{i}) + l_G(a_{1},a'_{1})\\&\quad +2 l_G(a_{1},b_{1})+ 2l_G(a_{1},c_{1})\\&= \sum \limits _{i=2}^{\frac{n+1}{2}} l_G(a_{1},a_{i})+\sum \limits _{i=\frac{n+3}{2}}^{n} l_G(a_{1},a_{i})+2\Bigg  [\sum \limits _{i=2}^{\frac{n+1}{2}} l_G(a_{1},b_{i})+\sum \limits _{i=\frac{n+3}{2}}^{n} l_G(a_{1},b_{i})\Bigg  ]\\&\quad +2\Bigg  [\sum \limits _{i=2}^{\frac{n+1}{2}} l_G(a_{1},c_{i}) + \sum \limits _{i=\frac{n+3}{2}}^{n} l_G(a_{1},c_{i})\Bigg  ] \\&\quad + \sum \limits _{i=2}^{\frac{n-1}{2}} l_G(a_{1},a'_{i}) +\sum \limits _{i=\frac{n+1}{2}}^{n} l_G(a_{1},a'_{i}) + l_G(a_{1},a'_{1})+2 l_G(a_{1},b_{1})+ 2l_G(a_{1},c_{1})\\&= \sum \limits _{i=2}^{\frac{n+1}{2}} 4(n-i+1) +\sum \limits _{i=\frac{n+3}{2}}^{n} 4(i-1)+2\Bigg  [ \sum \limits _{i=2}^{\frac{n+1}{2}} [4(n-i)+5] + \sum \limits _{i=\frac{n+3}{2}}^{n} (4i+1)\Bigg  ] \\&\quad + 2\Bigg  [ \sum \limits _{i=2}^{\frac{n+1}{2}} [4(n-i)+6] + \sum \limits _{i=\frac{n+3}{2}}^{n} 4i\Bigg  ]\\&\quad +\sum \limits _{i=2}^{\frac{n-1}{2}} [4(n-i)+1]+\sum \limits _{i=\frac{n+1}{2}}^{n} (4i-1) +(4n-3)+2(4n-1)+2(4n-2)\\&= 18n^2+8n-13. \end{aligned}$$

For *n* even, the detour transmission of $b_1 \in V(G)$

$$\begin{aligned} \omega (b_{1})&= \sum \limits _{x \in V(G)} l_G(b_{1},x)\\&= \sum \limits _{i=1}^{n} l_G(b_{1},a_{i}) +\sum \limits _{i=2}^{n} l_G(b_{1},b_{i}) + \sum \limits _{i=1}^{n} l_G(b_{1},c_{i}) + \sum \limits _{i=1}^{n} l_G(b_{1},a'_{i}) +\sum \limits _{i=1}^{n} l_G(b_{1},b'_{i}) + \sum \limits _{i=1}^{n} l_G(b_{1},c'_{i})\\&= \sum \limits _{i=2}^{n} l_G(b_{1},a_{i})+2 \sum \limits _{i=2}^{n} l_G(b_{1},b_{i})\\&\quad +2 \sum \limits _{i=2}^{n} l_G(b_{1},c_{i}) + \sum \limits _{i=2}^{n} l_G(b_{1},a'_{i}) +l_G(b_{1},a_{1})+l_G(b_{1},a'_{1})+ l_G(b_{1},b'_{1}) + 2l_G(b_{1},c_{1})\\&= \sum \limits _{i=2}^{\frac{n}{2}+1} l_G(b_{1},a_{i})+\sum \limits _{i=\frac{n}{2}+2}^{n} l_G(b_{1},a_{i})+2\Bigg  [ \sum \limits _{i=2}^{\frac{n}{2}} l_G(b_{1},b_{i})\\&\quad +\sum \limits _{i=\frac{n}{2}+1}^{n} l_G(b_{1},b_{i})\Bigg  ] + 2\Bigg  [ \sum \limits _{i=2}^{\frac{n}{2}+1} l_G(b_{1}, c_{i}) +\sum \limits _{i=\frac{n}{2}+2}^{n} l(b_{1},c_{i})\Bigg  ] + \sum \limits _{i=2}^{\frac{n}{2}} l_G(b_{1},a'_{i}) \\&\quad + \sum \limits _{i=\frac{n}{2}+1}^{n} l_G(b_{1},a'_{i})+l_G(b_{1},a_{1})+l_G(b_{1},a'_{1})+ l_G(b_{1},b'_{1}) + 2l_G(b_{1},c_{1})\\&= \sum \limits _{i=2}^{\frac{n}{2}+1} [4(n-i)+9] +\sum \limits _{i=\frac{n}{2}+2}^{n} (4i-3)+2\Bigg  [ \sum \limits _{i=2}^{\frac{n}{2}} [4(n-i)+10] +\sum \limits _{i=\frac{n}{2}+1}^{n} (4i+2)\Bigg  ] \\&\quad + 2\Bigg  [\sum \limits _{i=2}^{\frac{n}{2}+1} [4(n-i)+11] + \sum \limits _{i=\frac{n}{2}+2}^{n} (4i+1)\Bigg  ]\\&\quad + \sum \limits _{i=2}^{\frac{n}{2}} [4(n-i)+6]+ \sum \limits _{i=\frac{n}{2}+1}^{n} 4i +(4n-1)+(4n-2)+4n+2(4n-1)\\&= 18n^2+26n-30. \end{aligned}$$

For *n* is odd,

$$\begin{aligned} \omega (b_{1})&= \sum \limits _{x \in V(G)} l_G(b_{1},x)\\&= \sum \limits _{i=1}^{n} l_G(b_{1},a_{i}) +\sum \limits _{i=2}^{n} l_G(b_{1},b_{i}) + \sum \limits _{i=1}^{n} l_G(b_{1},c_{i}) + \sum \limits _{i=1}^{n} l_G(b_{1},a'_{i}) \\&\quad +\sum \limits _{i=1}^{n} l_G(b_{1},b'_{i}) + \sum \limits _{i=1}^{n} l_G(b_{1},c'_{i})\\&= \sum \limits _{i=2}^{n} l_G(b_{1},a_{i})+2 \sum \limits _{i=2}^{n} l_G(b_{1},b_{i}) +2 \sum \limits _{i=2}^{n} l_G(b_{1},c_{i}) + \sum \limits _{i=2}^{n} l_G(b_{1},a'_{i})\\&\quad +l_G(b_{1},a_{1})+l_G(b_{1},a'_{1})+ l_G(b_{1},b'_{1})+ + 2 l_G(b_{1},c_{1})\\&= \sum \limits _{i=2}^{\frac{n+1}{2}} l_G(b_{1},a_{i})+\sum \limits _{i=\frac{n+3}{2}}^{n} l_G(b_{1},a_{i})\\&\quad +2\Bigg  [ \sum \limits _{i=2}^{\frac{n+1}{2}} l_G(b_{1},b_{i})+\sum \limits _{i=\frac{n+3}{2}}^{n} l_G(b_{1},b_{i})\Bigg  ] \\&\quad +2\Bigg  [\sum \limits _{i=2}^{\frac{n+1}{2}} l_G(b_{1},c_{i}) + \sum \limits _{i=\frac{n+3}{2}}^{n} l_G(b_{1},c_{i})\Bigg  ] \\&\quad + \sum \limits _{i=2}^{\frac{n+1}{2}} l_G(b_{1},a'_{i})+ \sum \limits _{i=\frac{n+3}{2}}^{n} l_G(b_{1},a'_{i})+l_G(b_{1},a_{1})+ l_G(b_{1},a'_{1}) + l_G(b_{1},b'_{1})+ 2l_G(b_{1},c_{1})\\&= \sum \limits _{i=2}^{\frac{n+1}{2}} [4(n-i)+9] +\sum \limits _{i=\frac{n+3}{2}}^{n}(4i-3)+2\Bigg  [ \sum \limits _{i=2}^{\frac{n+1}{2}} [4(n-i)+10] +\sum \limits _{i=\frac{n+3}{2}}^{n} (4i+2) \Bigg  ] \\&\quad + 2\Bigg  [ \sum \limits _{i=2}^{\frac{n+1}{2}} [4(n-i)+11)] +\sum \limits _{i=\frac{n+3}{2}}^{n} (4i+1)\Bigg  ]\\&\quad +\sum \limits _{i=2}^{\frac{n+1}{2}} [4(n-i)+6] +\sum \limits _{i=\frac{n+3}{2}}^{n} 4i +(4n-1)+(4n-2)+4n+2(4n-1)\\&= 18n^2+26n-29. \end{aligned}$$

$$\square $$

### **Theorem 1**

*Let* *G* *be a molecular graph of cycloparaphenylene of dimension* *n*. *Then*

$$\begin{aligned} \omega (G)=\left\{ \begin{array}{ll} 54n^3+60n^2-72n &{} \hbox {if }n\hbox { is even} \\ 54n^3+60n^2-71n &{} \hbox {if }n\hbox { is odd} \end{array} \right. \end{aligned}$$

### **Proof**

Let *n* be even

Due to symmetry $\omega (x)=\omega (x')$, where $x\in \{a_i,b_i,c_i\}$ and also $\omega (b_i)=\omega (c_i)$. Now,

$$\begin{aligned} \omega (G)&={\frac{1}{2}}\sum \limits _{u\in V(G)}\omega (u)\\ &={\frac{1}{2}}\Bigg  [2n\omega (a_1)+4n\omega (b_1)\Bigg  ] \\ &=n\omega (a_1)+2n\omega (b_1) \\ &=n(18n^2+8n-12)+2n(18n^2+26n-30) \\ \omega (G)&=54n^3+60n^2-72n. \end{aligned}$$

with the similar argument along with Lemma 4, we derive the result for *n* odd.

$$\square $$

### **Lemma 5**

*Let* *G* *be a molecular graph of a linear polyphenylene of dimension* *n*, *and* $\{a_i,a_i',b_i,b_i',c_i,c_i': 1 \le i \le n\}$ *be the vertex set of* *G*. *Then*,

(i) $$\omega (a_i)=24i^2-42i+12n^2-24ni+33n+18.$$
(ii) $$\omega (b_i) = 24i^2-18i+12n^2-24ni+39n-12.$$
(iii) $$\omega (c_i)= 24i^2-30i+12n^2-24ni+45n-6.$$
(iv) $$\omega (a'_{i})= 24i^2-6i+12n^2-24ni+15n.$$

### Proof

For any *n* and $a_i \in V(G)$, the detour transmission of $a_i$ is given by

$$\begin{aligned} \omega (a_{i})&= \sum \limits _{x\in V(G)}l_G(a_i,x)\\&= \sum \limits _{j=1}^{n} l_G(a_{i},a_{j}) +\sum \limits _{j=1}^{n} l_G(a_{i},b_{j}) + \sum \limits _{j=1}^{n} l_G(a_{i},c_{j}) + \sum \limits _{j=1}^{n} l_G(a_{i},a'_{j}) +\sum \limits _{j=1}^{n} l_G(a_{i},b'_{j}) + \sum \limits _{j=1}^{n} l_G(a_{i},c'_{j})\\&=\sum \limits _{j=1}^{i-1} l_G(a_{i},a_{j})+\sum \limits _{j=i+1}^{n} l_G(a_{i},a_{j})+ \sum \limits _{j=1}^{i-1} l_G(a_{i},b_{j})+l_G(a_{i},b_{i})+ \sum \limits _{j=i+1}^{n} l_G(a_{i},b_{j})+ \sum \limits _{j=1}^{i-1} l_G(a_{i},c_{j})+l_G(a_{i},c_{i}) \\&\quad + \sum \limits _{j=i+1}^{n}l_G(a_{i},c_{j}) + \sum \limits _{j=1}^{i-1} l_G(a_{i},a'_{j})+l_G(a_{i},a'_{i})+\sum \limits _{j=i+1}^{n} l_G(a_{i},a'_{j})+\sum \limits _{j=1}^{i-1} l_G(a_{i},b'_{j}) + l_G(a_{i},b'_{i}) \\&\quad +\sum \limits _{j=i+1}^{n} l_G(a_{i},b'_{j})+\sum \limits _{j=1}^{i-1} l_G(a_{i},c'_{j})+l_G(a_{i},c'_{i}) +\sum \limits _{j=i+1}^{n} l_G(a_{i},c'_{j})\\&=\sum \limits _{j=1}^{i-1} l_G(a_{i},a_{j})+\sum \limits _{j=i+1}^{n} l_G(a_{i},a_{j})+2 \sum \limits _{j=1}^{i-1} l_G(a_{i},b_{j})+2 \sum \limits _{j=i+1}^{n} l_G(a_{i},b_{j}) + 2\sum \limits _{j=1}^{i-1} l_G(a_{i},c_{j}) + 2 \sum \limits _{j=i+1}^{n} l_G(a_{i},c_{j}) \\&\quad + \sum \limits _{j=1}^{i-1} l_G(a_{i},a'_{j}) +\sum \limits _{j=i+1}^{n} l_G(a_{i},a'_{j})+ l_G(a_{i},b_{i})+ l_G(a_{i},c_{i}) + l_G(a_{i},a'_{i})+l_G(a_{i},b'_{i})+l_G(a_{i},c'_{i})\\&=\sum \limits _{j=1}^{i-1} 4(i-j)+\sum \limits _{j=i+1}^{n} 4(j-i)+2\Bigg  [ \sum \limits _{j=1}^{i-1} [4(i-j)+1]+ \sum \limits _{j=i+1}^{n} [4(j-i)+5]\Bigg  ] + 2\Bigg  [\sum \limits _{j=1}^{i-1} [4(j-i)+2] \\&\quad + \sum \limits _{j=i+1}^{n} [4(j-i)+4] \Bigg  ]+\sum \limits _{j=1}^{i-1} [4(i-j-1)+1] + \sum \limits _{j=i+1}^{n} [4(j-i)+3]+5+4+3+5+4 \\&=\sum \limits _{j=1}^{i-1} [20(i-j)+6]+\sum \limits _{j=i+1}^{n}[24(j-i)+21]+ \sum \limits _{j=1}^{i-1}[ 4(i-j-1)+1] +21 \\&= 24i^2-42i+12n^2-24ni+33n+18. \end{aligned}$$

For $b_i \in V(G)$, the detour transmission of $b_i$ is given by

$$\begin{aligned} \omega (b_{i})&= \sum \limits _{x\in V(G)}l_G(b_i,x)\\&= \sum \limits _{j=1}^{n} l_G(b_{i},a_{j}) +\sum \limits _{j=1}^{n} l_G(b_{i},b_{j}) + \sum \limits _{j=1}^{n} l_G(b_{i},c_{j}) + \sum \limits _{j=1}^{n} l_G(b_{i},a'_{j}) +\sum \limits _{j=1}^{n} l_G(b_{i},b'_{j}) + \sum \limits _{j=1}^{n} l_G(b_{i},c'_{j})\\&=\sum \limits _{j=1}^{i-1} l_G(b_{i},a_{j})+l_G(b_{i},a_{i})+\sum \limits _{j=i+1}^{n} l_G(b_{i},a_{j})+ \sum \limits _{j=1}^{i-1} l_G(b_{i},b_{j})+ \sum \limits _{j=i+1}^{n} l_G(b_{i},b_{j})+ \sum \limits _{j=1}^{i-1} l_G(b_{i},c_{j}) + l_G(b_{i},c_{i}) \\&\quad +\sum \limits _{j=i+1}^{n}l_G(b_{i},c_{j}) + \sum \limits _{j=1}^{i-1} l_G(b_{i},a'_{j})+l_G(b_{i},a'_{i})+\sum \limits _{j=i+1}^{n} l_G(b_{i},a'_{j})+\sum \limits _{j=1}^{i-1} l_G(b_{i},b'_{j})+ l_G(b_{i},b'_{i})+\sum \limits _{j=i+1}^{n} l_G(b_{i},b'_{j}) \\&\quad + \sum \limits _{j=1}^{i-1} l_G(b_{i},c'_{j})+l_G(b_{i},c'_{i}) +\sum \limits _{j=i+1}^{n} l_G(b_{i},c'_{j})\\&=\sum \limits _{j=1}^{i-1} l_G(b_{i},a_{j})+\sum \limits _{j=i+1}^{n} l_G(b_{i},a_{j})+2 \Bigg  [\sum \limits _{j=1}^{i-1} l_G(b_{i},b_{j})+ \sum \limits _{j=i+1}^{n} l_G(b_{i},b_{j})\Bigg  ] + 2\Bigg  [ \sum \limits _{j=1}^{i-1} l_G(b_{i},c_{j}) + 2 \sum \limits _{j=i+1}^{n} l_G(b_{i},c_{j})\Bigg  ] \\&\quad +\sum \limits _{j=1}^{i-1} l_G(b_{i},a'_{j})+\sum \limits _{j=i+1}^{n} l_G(b_{i},a'_{j})+ l_G(b_{i},a_{i})+ l_G(b_{i},c_{i}) + l_G(b_{i},a'_{i})+l_G(b_{i},b'_{i})+l_G(b_{i},c'_{i})\\&=\sum \limits _{j=1}^{i-1} [4(i-j)+5]+\sum \limits _{j=i+1}^{n} [4(j-i)+1]+2\Bigg  [ \sum \limits _{j=1}^{i-1} [4(i-j)+6]+ \sum \limits _{j=i+1}^{n} [4(j-i)+6]\Bigg  ]&\\&\ \ \ + 2\Bigg  [\sum \limits _{j=1}^{i-1} [4(i-j)+7] + \sum \limits _{j=i+1}^{n} [4(j-i)+5]\Bigg  ] +\sum \limits _{j=1}^{i-1} [4(i-j-1)+6] + \sum \limits _{j=i+1}^{n} [4(j-i)+4]+21 \\&=\sum \limits _{j=1}^{i-1} [20(i-j)+31]+\sum \limits _{j=i+1}^{n}[24(j-i)+27]+ \sum \limits _{j=1}^{i-1} [4(i-j-1)+6] +21\\&= 24i^2-18i+12n^2-24ni+39n-12. \end{aligned}$$

And for $c_i \in V(G)$, the detour transmission of $c_i$ is given by

$$\begin{aligned} \omega (c_{i})&= \sum \limits _{x\in V(G)}l_G(c_i,x)\\&= \sum \limits _{j=1}^{n} l_G(c_{i},a_{j}) +\sum \limits _{j=1}^{n} l_G(c_{i},b_{j}) + \sum \limits _{j=1}^{n} l_G(c_{i},c_{j}) + \sum \limits _{j=1}^{n} l_G(c_{i},a'_{j}) +\sum \limits _{j=1}^{n} l_G(c_{i},b'_{j}) + \sum \limits _{j=1}^{n} l_G(c_{i},c'_{j})\\&=\sum \limits _{j=1}^{i-1} l_G(c_{i},a_{j})+l_G(c_{i},a_{i})+\sum \limits _{j=i+1}^{n} l_G(c_{i},a_{j})+ \sum \limits _{j=1}^{i-1} l_G(c_{i},b_{j})+l_G(c_{i},b_{i})+ \sum \limits _{j=i+1}^{n} l_G(c_{i},b_{j})\\&\quad + \sum \limits _{j=1}^{i-1} l_G(c_{i},c_{j}) + \sum \limits _{j=i+1}^{n}l_G(c_{i},c_{j}) +\sum \limits _{j=1}^{i-1} l_G(c_{i},a'_{j})+l_G(c_{i},a'_{i})+\sum \limits _{j=i+1}^{n} l_G(c_{i},a'_{j})\\&\quad +\sum \limits _{j=1}^{i-1} l_G(c_{i},b'_{j}) + l_G(c_{i},b'_{i})+\sum \limits _{j=i+1}^{n} l_G(c_{i},b'_{j}) \\&\quad +\sum \limits _{j=1}^{i-1} l_G(c_{i},c'_{j})+l_G(c_{i},c'_{i}) +\sum \limits _{j=i+1}^{n} l_G(c_{i},c'_{j})\\&=\sum \limits _{j=1}^{i-1} l_G(c_{i},a_{j})+\sum \limits _{j=i+1}^{n} l_G(c_{i},a_{j})+2 \Bigg  [\sum \limits _{j=1}^{i-1} l_G(c_{i},b_{j})+ \sum \limits _{j=i+1}^{n} l_G(c_{i},b_{j})\Bigg  ] + 2\Bigg  [ \sum \limits _{j=1}^{i-1} l_G(c_{i},c_{j}) + \sum \limits _{j=i+1}^{n} l_G(c_{i},c_{j})\Bigg  ] \\&\quad + \sum \limits _{j=1}^{i-1} l_G(c_{i},a'_{j}) +\sum \limits _{j=i+1}^{n} l_G(c_{i},a'_{j})+ l_G(c_{i},a_{i}) + l_G(c_{i},a'_{i})+l_G(c_{i},b_{i})+l_G(c_{i},b'_{i})+l_G(c_{i},c'_{i})\\&=\sum \limits _{j=1}^{i-1} [4(i-j)+4]+\sum \limits _{j=i+1}^{n} [4(j-i)+2]+2\Bigg  [ \sum \limits _{j=1}^{i-1}[ 4(i-j)+5]+ \sum \limits _{j=i+1}^{n} [4(j-i)+7]\Bigg  ] \\&\quad + 2\Bigg  [\sum \limits _{j=1}^{i-1} [4(i-j)+6]+ \sum \limits _{j=i+1}^{n} [4(j-i)+6]\Bigg  ] +\sum \limits _{j=1}^{i-1} [4(i-j-1)+5] + \sum \limits _{j=i+1}^{n} [4(j-i)+5]+21 \\&=\sum \limits _{j=1}^{i-1} [20(i-j)+26]+\sum \limits _{j=i+1}^{n}[24(j-i)+33]+ \sum \limits _{j=1}^{i-1}[ 4(i-j-1)+5] +21\\&= 24i^2-30i+12n^2-24ni+45n-6. \end{aligned}$$

Now for $a_i' \in V(G)$, the detour transmission of a vertex $a_i'$ is

$$\begin{aligned} \omega (a'_{i})&= \sum \limits _{x\in V(G)}l_G(a'_i,x)\\&= \sum \limits _{j=1}^{n} l_G(a'_{i},a_{j}) +\sum \limits _{j=1}^{n} l_G(a'_{i},b_{j}) + \sum \limits _{j=1}^{n} l_G(a'_{i},c_{j}) + \sum \limits _{j=1}^{n} l_G(a'_{i},a'_{j}) +\sum \limits _{j=1}^{n} l_G(a'_{i},b'_{j}) + \sum \limits _{j=1}^{n} l_G(a'_{i},c'_{j})\\&=\sum \limits _{j=1}^{i-1} l_G(a'_{i},a_{j})+l_G(a'_{i},a_{i})+\sum \limits _{j=i+1}^{n} l_G(a'_{i},a_{j})+ \sum \limits _{j=1}^{i-1} l_G(a'_{i},b_{j})+l_G(a'_{i},b_{i})\\&\quad + \sum \limits _{j=i+1}^{n} l_G(a'_{i},b_{j})+ \sum \limits _{j=1}^{i-1} l_G(a'_{i},c_{j}) \\&\quad + l_G(a'_{i},c_{i}) +\sum \limits _{j=i+1}^{n}l_G(a'_{i},c_{j})+ \sum \limits _{j=1}^{i-1} l_G(a'_{i},a'_{j})+\sum \limits _{j=i+1}^{n} l_G(a'_{i},a'_{j})\\&\quad +\sum \limits _{j=1}^{i-1} l_G(a'_{i},b'_{j}) + l_G(a'_{i},b'_{i})+\sum \limits _{j=i+1}^{n} l_G(a'_{i},b'_{j}) \\&\quad +\sum \limits _{j=1}^{i-1} l_G(a'_{i},c'_{j}) +l_G(a'_{i},c'_{i})+\sum \limits _{j=i+1}^{n} l_G(a'_{i},c'_{j})\\&=\sum \limits _{j=1}^{i-1} l_G(a'_{i},a_{j})+\sum \limits _{j=i+1}^{n} l_G(a'_{i},a_{j})+2 \sum \limits _{j=1}^{i-1} l_G(a'_{i},b_{j})+2 \sum \limits _{j=i+1}^{n} l_G(a'_{i},b_{j}) + 2\sum \limits _{j=1}^{i-1} l_G(a'_{i},c_{j}) + 2 \sum \limits _{j=i+1}^{n} l_G(a'_{i},c_{j}) \\&\quad + \sum \limits _{j=1}^{i-1} l_G(a'_{i},a'_{j})+\sum \limits _{j=i+1}^{n} l_G(a'_{i},a'_{j})+ l_G(a'_{i},a_{i})+l_G(a'_{i},b_{i})+l_G(a'_{i},c_{i})+l_G(a'_{i},b'_{i})+l_G(a'_{i},c'_{i})\\&=\sum \limits _{j=1}^{i-1} [4(i-j)+3]+\sum \limits _{j=i+1}^{n} [4(j-i-1)+1]+2\Bigg  [ \sum \limits _{j=1}^{i-1} [4(i-j)+4]+ \sum \limits _{j=i+1}^{n} [4(j-i-1)+6]\Bigg  ]+21 \\&\quad + 2\Bigg  [\sum \limits _{j=1}^{i-1} [4(i-j)+5]+ \sum \limits _{j=i+1}^{n} [4(j-i-1)+5]\Bigg  ] +\sum \limits _{j=1}^{i-1} [4(i-j-1)+4] + \sum \limits _{j=i+1}^{n} [4(j-i-1)+4] \\&=\sum \limits _{j=1}^{i-1} [20(i-j)+21]+\sum \limits _{j=i+1}^{n}[24(j-i-1)+27]+ \sum \limits _{j=1}^{i-1} [4(i-j-1)+4] +21\\&=24i^2-6i+12n^2-24ni+15n. \end{aligned}$$

$$\square $$

### **Theorem 2**

*Let* *G* *be a molecular graph of linear polyphenylene of dimension* *n*. *Then* $\omega (G)=24n^3+72n^2-33$.

### **Proof**

Due to symmetry, for any $b_i, c_i\in V(G)$, we have $\omega (b_i)=\omega (b'_i)$ and $\omega (c_i)=\omega (c'_i)$, and

$$\begin{aligned} \omega (G)&={\frac{1}{2}}\sum \limits _{u\in V(G)}\omega (u)\\&={\frac{1}{2}}\Bigg  [\sum \limits _{i=1}^{n} \omega (a_{i})+2\sum \limits _{i=1}^{n} \omega (b_{i})+2\sum \limits _{i=1}^{n} \omega (c_{i})+\sum \limits _{i=1}^{n} \omega _G(a'_{i})\Bigg  ] \\&={\frac{1}{2}}\Bigg  [\sum \limits _{i=1}^{n}(24i^2-42i+12n^2-24ni+33n+18)+ 2 \sum \limits _{i=1}^{n}(24i^2-18i+12n^2-24ni+39n-12) \\&\quad +2 \sum \limits _{i=1}^{n}(24i^2-30i+12n^2-24ni+45n-6) + \sum \limits _{i=1}^{n}(24i^2-6i+12n^2-24ni+15n)\Bigg  ] \\&=24n^3+72n^2-33. \end{aligned}$$

$$\square $$

**Figure 12:**
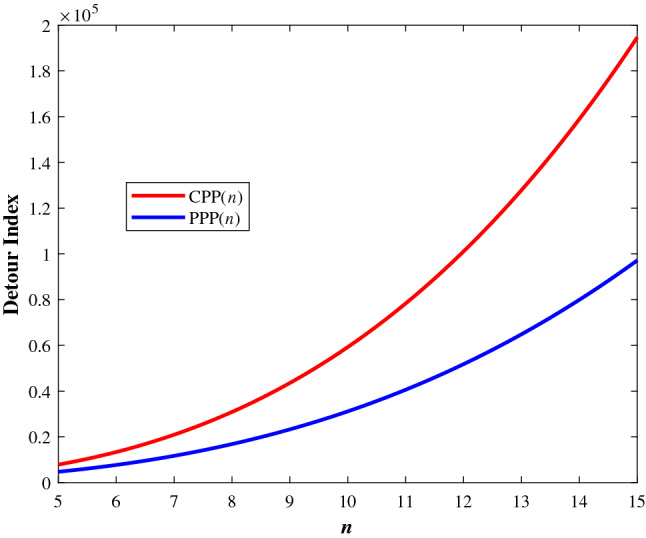
A comparative graphical representation of detour index of cycloparaphenylene CPP(*n*) and poly (*p*-phenylene) PPP(*n*).

The graphical representation of the detour index of cycloparaphenylene CPP(*n*) and poly (*p*-phenylene) PPP(*n*) were depicted in Fig. 12, which says that the detour index of cycloparaphenylene is higher than polyphenylene irrespective of *n*.

## Conclusion

In recent decade, CPPs have gone from being manufactured interests to promptly open materials with exceptionally tunable properties. The syntheses of CPPs are motivated by a wide extent of energizing applications, going from strong state nanomaterials to organic imaging. Also, the aromatic polymers of PPPs comprising of straightforwardly repeating benzene units as their spine. PPP has interesting optical properties, for example, electroluminescence, and is regularly utilized as tunable blue-transmitting material for light-radiating devices. Detour index is a promising topological index and the study of this index is very helpful to acquire the basic topologies of networks. We accept that the detour index acquired here well correspond with a portion of the physico-chemical properties and a portion of the structure-property relations.
